# Monitoring of a Productive Blue-Green Roof Using Low-Cost Sensors

**DOI:** 10.3390/s23249788

**Published:** 2023-12-12

**Authors:** Afsana Alam Akhie, Darko Joksimovic

**Affiliations:** Department of Civil Engineering, Toronto Metropolitan University, 350 Victoria St., Toronto, ON M5B 2K3, Canada; darkoj@torontomu.ca

**Keywords:** blue-green roof, rooftop farming, low-cost sensors, crop growth monitoring, thermal performance

## Abstract

Considering the rising concern over climate change and the need for local food security, productive blue-green roofs (PBGR) can be an effective solution to mitigate many relevant environmental issues. However, their cost of operation is high because they are intensive, and an economical operation and maintenance approach will render them as more viable alternative. Low-cost sensors with the Internet of Things can provide reliable solutions to the real-time management and distributed monitoring of such roofs through monitoring the plant as well soil conditions. This research assesses the extent to which a low-cost image sensor can be deployed to perform continuous, automated monitoring of a urban rooftop farm as a PBGR and evaluates the thermal performance of the roof for additional crops. An RGB-depth image sensor was used in this study to monitor crop growth. Images collected from weekly scans were processed by segmentation to estimate the plant heights of three crops species. The devised technique performed well for leafy and tall stem plants like okra, and the correlation between the estimated and observed growth characteristics was acceptable. For smaller plants, bright light and shadow considerably influenced the image quality, decreasing the precision. Six other crop species were monitored using a wireless sensor network to investigate how different crop varieties respond in terms of thermal performance. Celery, snow peas, and potato were measured with maximum daily cooling records, while beet and zucchini showed sound cooling effects in terms of mean daily cooling.

## 1. Introduction

The reduction in vegetative land and its replacement with asphalt and concrete are contributing to the rapid growth of warmer temperatures and urban heat island events in cities. Such developed and built environments are lowering the amount of greenery, and only a reduced amount of open space is available for green infrastructure and urban farming. There is an escalating concern about constant municipal developments in recent years, that has sparked advocacy for green, blue-green roofs and urban farming practices as effective strategies for stormwater management, climate change mitigation, and a reduction in carbon footprint through local food production. Green roofs (GRs) can be implemented in three most common forms: extensive, semi-intensive, and intensive [1,2,3,4]. Extensive roofs often have light and shallow media, with plants and greens requiring minimal equipment and upkeep, making these the least expensive in terms of installation and management. Semi-intensive roofs can include some large shrubs and trees but are primarily comprised of smaller plants. Alternatively, intensive roofs involve massive infrastructures in terms of roof configuration, such as deep media, access to irrigation, and runoff drainage systems. Intensive green roofs can support more diverse plant combinations and arrangements than the extensive version and can be utilized for farming and food production. The thick substrate supplies nutrients that foster the proper growth of plants. Intensive green roofs also have more remarkable water retention ability, but their application is less than extensive ones because of high maintenance and operation costs [5].

Over the past few years, green roofs have been constructed with extended drainage layers to increase their water retention capacity, making them blue-green roofs (BGRs) [6,7]. However, the number of these installations is still relatively small, and research is just beginning to examine their potential advantages. The additional drainage layer can retain more stormwater compared to a typical green roof [8,9]. The roof offers numerous advantages, including stormwater control, lowering the urban heat island effect, and energy conservation. It also supplies the vegetative layer with water through capillary rises, reducing the frequency of irrigation needs. BGRs are envisioned to strengthen the practice of urban farming by growing a variety of fruits and vegetables, and helping manage stormwater as well as urban heat island effects as a cumulative response to urban food crises and climatic change. As the shortage of arable land and water resources is rising, shifting towards BGRs will soon become ever more attractive and needed around the world.

Producing crops on blue-green roofs, suggested as a productive blue-green roof (PBGR) [10], is a relatively new concept compared to former rooftop farming that has been practiced for several decades in potted containers, greenhouses, and open-air rooftop urban farms. PBGRs could be an effective way to mitigate many environmental issues on their own, especially considering the rising concern over stormwater management and climate change, like GRs or BGRs. Moreover, farming supports the local food supply and contributes to a lesser carbon footprint. The productive roof systems vary greatly from sedum-planted roofs in that the vegetation layer is dynamic and changes significantly over the growing seasons. The most dynamic feature of PBGRs is the vegetative layer because it constantly evolves with the plant species. The vital components that can significantly affect how a PBGR grows and changes over time are its location, weather, soil media, plant species, and management practices. So, a PBGR should develop to a condition that meets its necessary functional criteria as it ages and expands its capacity when a variety of crop families are sown and harvested.

The most significant component of any GR’s design is the selection of plant species that increase its lifetime [11]. Similarly, plant diversity and dynamics on a PBGR contribute to its long-term stability and viability since their condition and growth impact PBGR functionality. Crop phenotypes can provide crucial information to consultants, allowing them to predict crop development and farming efficiency on a PBGR. A good indicator of phenotypes is plant height, which is frequently measured via intrusive and destructive approaches, disturbing healthy growth and necessitating laboratory analyses. Even portable and wearable sensors [12] might damage crops due to the complex stem and leaf structure of plant foliage [13,14]. Hence, nondestructive methods of plant phenotyping have emerged as a critical area of research in precision farming. Since crops vary from a few centimeters to one or two meters in height, the thermal efficiency of a PBGR might be significantly impacted by the choice of crops. Investigating the thermal benefits and interactions between the crops and the BGR system is critical as these two elements might offer even more significant environmental advantages if combined.

Toronto has over seven hundred green roofs, and more are anticipated to be added in future. The operation and maintenance of these green roofs and farms are about to become challenging for administrators to ensure the expected benefits and commercially manage them. Low-cost sensor technologies can provide reliable solutions by integrating real-time management practices in the distributed smart monitoring of such roofs. This study has been conducted in a PBGR by focusing on two key concerns and research questions: (1) whether a low-cost depth image sensor can be deployed to provide continuous and automated monitoring capabilities of the crops, and (2) does high-resolution monitoring aid in understanding the rooftop thermal performance of different crop varieties. The study developed a convenient technique to monitor crop growth nondestructively and explored the operational efficacy of an RGB-depth image sensor on a functional urban rooftop farm. An IoT-enabled system is deployed to collect data through low-cost wireless temperature and soil moisture sensors for crop-based thermal performance evaluations.

## 2. Literature Review

Rooftop farms have substantial operating costs since they are labor-intensive and typically need one full-time farm coordinator. Low-cost sensors and Internet of Things (IoT) technologies have many potentials to integrate affordable and resilient management practices and provide reliable smart monitoring solutions. IoT facilitates remote monitoring and operation, requires minimal energy, automatically records large volumes of data, may be controlled remotely, and improves efficiency [15,16,17]. IoT-based farming can promote agricultural automation by equipping it with a sensor network [18], which could increase crop yield and reduce operating costs [19]. During the crop growth period, observations and analyses can be conducted even in poor weather conditions when routine checks are not feasible. Low-cost edge devices can lessen human engagement with the physical environment, and their accessible cloud-based platforms can present significant data insights [20]. Minimizing physical activity on the rooftop farm will also eliminate the risk of crop and soil damage or other implications. The miniaturization and rapid manufacturing of electronic devices has also enriched the growing interest in sensor network research [21].

In recent years, the IoT has substantially made the monitoring of green roof performance simpler [22,23]. The moisture content, temperature, and humidity from various locations can be monitored to quantify the thermal performance of roofs. IoT has been used in research and agriculture to maximize crop yield and facilitate early diagnosis, real-time monitoring, production efficiency, and the management of water resources [16,24,25]. A sensor-equipped system can monitor and record the change in the environmental parameters in real-time and enable roof operators to respond quickly in critical conditions [26]. Wireless sensor networks (WSNs) can provide uninterrupted monitoring capabilities over a vast region by integrating multiple sensors and maintaining communication with data loggers [16,21]. End devices can use multiple frequency channels and data speeds for wireless communication with one or more gateways. The information can be accessed from the console using any smart device. 

Crop phenotyping can provide the knowledge required to determine whether crops grow effectively in a specific environment of a green/blue-green roof. Here, phenotype refers to a crop’s height, leaf area, biomass, and other distinguishing characteristics [27]. Experts and industrial growers rely on monitoring plant growth to maintain and grow more resilient and productive crops [28]. Phenotyping systems based on growth chambers and greenhouses offer the benefits of frequent test cycles and strict environmental control; but test methods are frequently confined by the pot size and the range of possible factors that may be tested [29]. To monitor crop growth, various investigations on nondestructive techniques have been investigated, some utilizing the 2D or 3D image-based system [24,30,31]. Stereo vision [32], LiDAR [33], multispectral [34,35,36,37,38], and hyperspectral [37,38,39,40] imaging have also been explored by the mentioned research and applications, although their expenses are quite high in terms of instrumentation costs. Depth cameras have also been recently used by studies [28,41,42,43] in laboratory settings to estimate plant height and measure leaf area, leaf length, or plant biomass. Different image sensors of diverse prices have been developed, which are being used in plant health monitoring research in recent years, be it RGB or infrared. They could be used to provide a convenient and profitable framework enabling a continuous plant health monitoring system, but the usage [17] and processing [24] of images at the edge devices of IoT networks have not yet been implemented successfully as a real-time crop monitoring tool. There are many commercial depth cameras available with various price ranges [44], but their operational performance varies significantly between indoor and outdoor settings [14,42,45]. The researchers that deployed various RGB-depth imaging sensors conducted their study in greenhouses or growth chambers using controlled light settings or black background to create ideal conditions.

While many imaging sensors have been studied for some years, using low-cost RGB-depth imagers is a relatively modern technology with numerous possibilities to explore. However, 2D imaging has several limitations such as inconsistency in the physical dimension and distance from the image sensor and the inability to fully extract 3D information due to complicated plant structures and overlying foliage when evaluated against 3D imaging [31]. Crop growth rate employing image segmentation algorithms renders potential, although experiments are often conducted in semi-commercial settings, emphasizing the importance of high-quality image settings [18]. It is essential to develop low-cost technology for image-based crop phenotyping on open rooftops. Recently, studies have been working to measure the field performances of some image sensors. Neupane et al. [44] evaluated eight depth cameras to assess their field performance and accuracy in measuring object distance. Since depth imagers routinely release new versions and decrease expansion after a while due to continued challenges, the study urged the regular assessment of new depth imagers.

An intensive roof enhances the thermal capacity of the green roof while further increasing its thermal resistance [46] by delaying the daily thermal peak [47]. Plants cultivated on the roof lessen rooftop temperature and improve the microclimate by absorbing heat, providing shade, reflecting sunlight, and through evapotranspiration. A BGR can bring down the roof surface temperature by up to 5 °C in the summertime [48]. However, PBGRs are still not a widespread practice like BGRs. The deep substrate and various plant species with different sizes and proportions create concerns regarding the expected thermal benefit. During summer, the high solar radiation and air temperature give rise to the rate of evaporation improving the cooling effect from PBGRs [49,50]. A sedum roof’s daily mean surface temperature can be 4 °C warmer compared to a vegetable section, making a productive roof a better alternative [51,52]. The prevalence of dense foliage, sealed canopy cover, and larger leaf areas are related to reduced surface temperatures during the day [53]. Depending on the form of the vegetation, day and night cooling can range from 0.3 °C to 1.0 °C and 0.0 °C to 2.0 °C in cities [54]. The plant leaves filter solar radiation by reflecting and scattering it. When the plant canopy is dense, more shade and cooling are available, lessening the solar radiation that reaches the soil substrate during the day [46].

Understanding PBGR microclimates is also essential for comprehending the cooling advantage they are anticipated to provide. The microclimate is the set of environmental factors that are monitored in specific locations in proximity to the surface. Temperature, humidity, and moisture content are some of the environmental factors that provide practical insights for choosing the vegetation type as well as operational facilities of rooftop farms. Most of the research based on thermal benefit and microclimate management in dry or wet periods is conducted involving extensive or small-scale green roofs, but very little research is available that has quantified intensive roof performance. Similar facts were acknowledged in [55,56], insisting on the need for more research on the thermal performance of full-scale green roofs where the temperature fluctuates abruptly within a short period.

## 3. Materials and Methods

The study was conducted in the Toronto Metropolitan University (TMU) Urban Farm Living Lab. Instead of using modular systems, this fully operational rooftop farm provides in-field crop monitoring opportunities to explore actual PBGR performance in terms of vegetative growth and cooling benefits. The roof area is approximately 1000 square meters (slope 1%). It was first established in 2014 as a conventional GR and later converted to a PBGR in 2019. First a waterproofing layer is placed on the roof to secure the roof and building from water damage. Then, an overlaying root barrier membrane shields the roof against invasive plant roots. A 60 mm drainage layer, comprising 50 mm of small retention basins and 10 mm of airspace, that can retain water for plant uptake and gently discharge it off the roof instead of draining off quickly is added on top. A fabric filter layer is added to prevent fine particles from clogging the drainage layer. The growing medium varies from 250 to 300 mm in depth depending upon the application of annual compost or biochar at the start of the growing season. Approximately 35 types of crops are harvested on the farm each season. Monitoring was conducted from June 2022 to the end of September 2022. A previous study [57] conducted on this specific BGR tested the growing medium and found it to be 15.4% organic content comprising 3.67% gravel, 96.31% sand, and 0.02% fine materials with a moisture-holding capacity higher than 90%.

For the monitoring of plant growth, three crops with varying heights were selected: okra (*Abelmoschus esculentus*), bush bean (*Phaseolus vulgaris*), and carrot (*Daucus carota*). The average standard-sized okra plant can usually grow up to 2.5 m tall in a warm and humid climate. The bean plants are small and bushy with an ultimate growth of fewer than 0.6 m after maturing. The carrot is a root vegetable with a thin leafy stem being less than 0.6 m in height, and it produces best in cooler temperatures. The growth monitoring started on 5th August with okra and bush bean, and the carrot was monitored during late September. Since the farm had to maintain a specific working schedule, the images were collected at different times of the day, and the interval of image scanning was not precisely one week. Okra plants were observed for eight weeks starting from 5th August to 23rd September, bush beans from 5th August to 15th September, and carrots from 7th September to 5th October.

For rooftop microclimate and thermal performance monitoring, a concrete pavement section of the roof was used as a control to assess the cooling effect. Crops that have been monitored for thermal performance were potato (*Solanum tuberosum*), celery (*Apium graveolens*), snow peas (*Pisum sativum var. saccharatum*), summer squash—zucchini (*Cucurbita pepo*), carrot (*Daucus carota*), and beets (*Beta vulgaris*). Among these, potatoes, carrots, and beets are root vegetables.

The names and families of the monitored crops are listed In Table 1.

While selecting the image sensor, preference was given to one with a light weight and lower price that can also provide the desired components to conduct the study. OAK D-Lite, an RGB-depth sensor created lately for commercial usage by its maker Luxonis, CO, USA [59], was used in this study. This 61-g RGB-depth sensor is 91 mm in length, 17.5 mm in width, and 28 mm thick. The device is accessible through a USB 3 Type-C to Type-A cable using hosts like a computer or Raspberry Pi. The imager has a Sony’s IMX214 image sensor for RGB with a 4208 × 3120 resolution in the center, and OmniVision OV7251 sensors with a 640 × 480 resolution for stereo vision pairs [59]. The pixel sizes of the RGB and mono cameras are 1.12 µm × 1.12 µm and 3 µm × 3 µm, respectively. A wheeled cart was built for the acquisition system that was pushed through the paths between the plant beds. The platform was developed to perform weekly scans of the plants in connection with a computer as Oak D-Lite required a central controller to retrieve data. A vertical steel pole was used and had extended horizontal steel attached to it with the ability of height and distance adjustment. In vertical imaging, the altitude of the camera varied between 165 cm and 210 cm for okra.

Long range wide area network (LoRaWAN) is an effective communication protocol [60] developed in recent years that connects edge devices and network gateways. A LoRaWAN sensor module sends a signal to a LoRaWAN gateway, which receives it and forwards this signal across the specific IoT cloud network. The gateways are an open channel for message transmission between the edge devices and a central network server in the background. The data, for example—temperature, is received from sensors and transmitted to The Things Network (TTN) console through the gateway. To monitor the thermal performance, the study used SenseCAP Wireless Air Temperature and Humidity Sensors (LoRa-S-915-TH, Seeed Studio) above the plant bed level at two different heights of 15 cm and 60 cm from the soil. Each of these sensors recorded the battery voltage, air temperature in degrees Celsius, and humidity in percentage every 15 min. The accuracy of the sensor is +/−0.2 °C and +/−1.5%RH [61].

**Figure 1:**
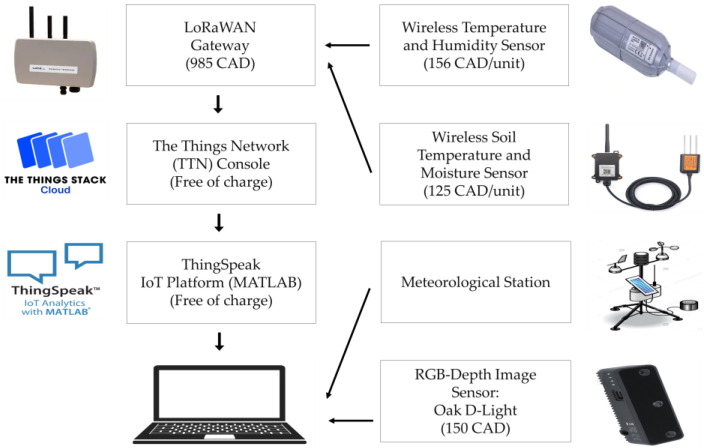
Sensor components and IoT architecture.

Ambient air temperature, relative humidity, dew point, wind speed, and photosynthetically active radiation (PAR) were measured using a meteorological station at a 2 m height on the roof. Dragino LSE01 LoRaWAN Soil Moisture and EC Sensors were used in the mid-depth of the substrate which measured substrate temperature (accuracy <0.3 °C) and volumetric moisture content (accuracy +/−3% for 0–53%, +/−5% for >53%) [62] at 10 min intervals. Before installing the sensors, they were calibrated in the lab using the same soil media. The dry bulk density of the soil was measured as 0.45 gm/cm3. All the sensors were registered on the TTN console using their distinct extended unique identifier (EUI) number and connected to webhook channels through theThingsSpeak IoT analytic platform. The webhook accepts the message sent by the end devices and forwards it to the corresponding channels. ThingsSpeak enables access to data logged into the channels from the webhook streaming, utilizing their unique channel ID. The architecture of the IoT monitoring system is shown in Figure 1.

**Figure 2:**
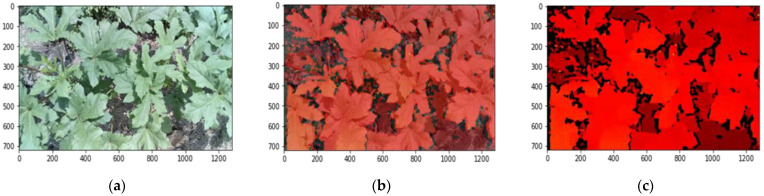
Images collected by Oak-D Lite for okra plants: (**a**) RGB image; (**b**) aligned and blended RGB-depth image; (**c**) aligned depth map.

The depth camera was deployed to collect RGB images with corresponding aligned RGB images, depth maps, and, most importantly, the disparity matrices from scans. The default system works in indoor settings in the absence of bright sunlight with specific exposure and iso settings. The system was modified to be used outdoors with auto exposure and autofocus options enabled for RGB cameras. The RGB camera and mono cameras were then programmed to generate aligned images to ensure that the depth map also aligns with the pixels of the RGB image according to their coordinates. The depth map was processed using the disparity that the stereo camera measures from the scene. The device captured an RGB image with a resolution of 1280 × 720, an upscaled blended image and a depth map, as shown in Figure 2, and the disparity matrices of the same number of cells as pixels. The distance between any two matching spots in a stereo pair’s left and right images is referred to as disparity. The disparity information is later used to find out the pixel-wise depth from the RGB image. Since the resolution of the stereo cameras was 640 × 480, the depth map and hence the disparity matrix were both upscaled to match the RGB resolution while storing the aligned blended image. The image acquisition system was developed using DepthAI API (v2.17.2.0) [63] in Python.

The retrieved RGB images were first cropped to remove the potential boundary regions with little to no disparity or interference created by the soil path or cart bottoms. The cropped images were then converted to grayscale and segmented using the global OTSU thresholding technique [64] in an automated approach developed in Python. Images were processed to segregate the leaf-constructing pixel coordinates from the rest of the image background. The resulting binary images had separate ground-level pixels as the foreground after applying a mask over the leaf pixels and included some areas with less brightness or no leaf region.

**Figure 3:**
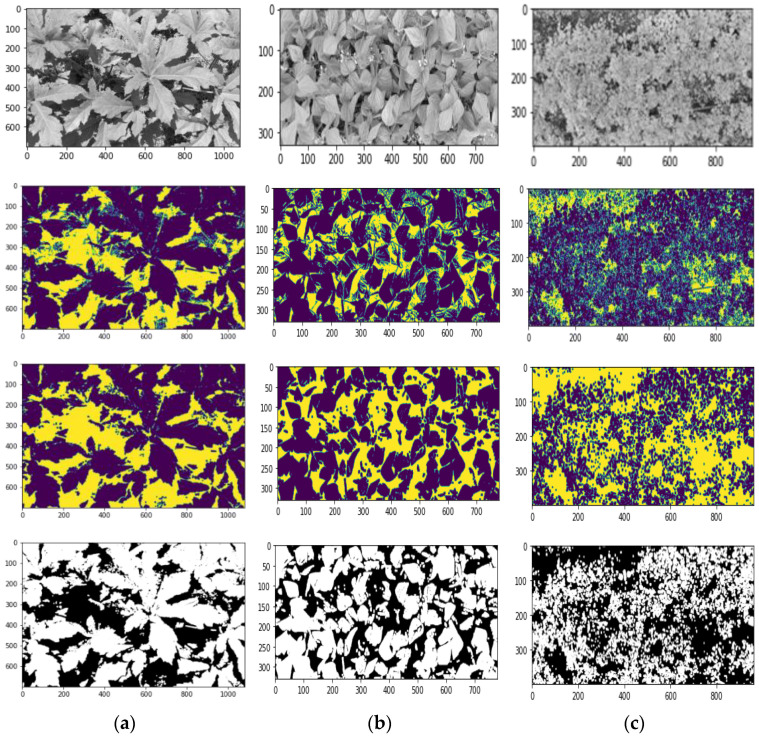
Grayscale image, OTSU segmentation, morphological operation, and final segmented images of (**a**) okra plants, (**b**) bush beans, and (**c**) carrot plants.

As images were taken from just above the plant canopy, they had areas constituting the irregularly shaded parts due to the neighbor leaves’ shadow, the inclined orientation of leaves spreading from the stem in random directions, sunlight coming from altered angles at different times, and soil from the ground level through the canopy holes. Many ground-forming pixels because of smaller leaves and slimmer stems were identified to be included in the leaf pixels regions generating noise, which were then effectively removed through a morphological closing and erosion operation [65]. This process was implemented through the opening or closing of the neighboring pixels of the detected foreground boundary, followed by erosion or dilation. The analysis was run using small (3 × 3) kernels. The quality of the segmented image and noise removal was confirmed by the reduction in the number of pixels that constructed the leaf area. For better visualization, the final output images were adjusted by setting the background (ground/soil) with RGB = 0 as black and the foreground (plant leaves) with RGB = 255 as white, as shown in Figure 3.

**Figure 4:**
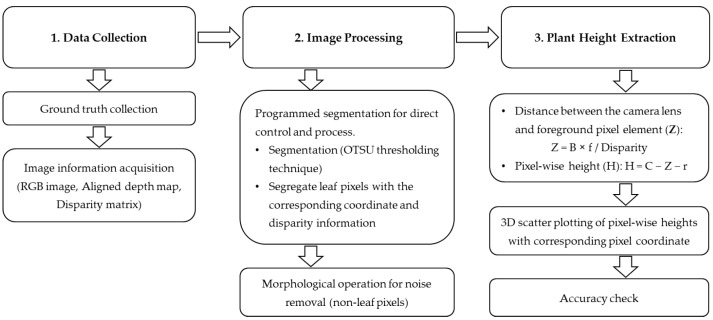
Workflow diagram of plant height determination.

After the segmentation and morphological operation, the separated foreground pixels were aligned with the disparity matrix based on their corresponding pixel coordinates. Once the coordinates were aligned, each pixel’s disparity was converted to depths (*Z*) as the distance from the camera center to the plant canopy and stored in several arrays according to weeks for further analysis. The distance between two matching points in the image plane that corresponds to the camera center and the scene points in three dimensions is referred to as disparity (*D*) in pixels. The disparity between matching image pixels and their corresponding depth in a scene is inversely related. The pixel-wise depth (*Z*) was found from the similar triangle rule as Equation (1), where *B* is the stereo camera distance or the baseline distance (7.5 cm for Oak D Lite), and *f* is the camera’s focal length in pixels determined beforehand during camera calibration. The pixel-wise plant height (*H*) is calculated as Equation (2), where *C* is the camera elevation at the field, and *r* is the bed rise from the ground. The representative plant height from the image (*IPH*) is then measured from the 95–100th percentile value of the heights among foreground pixels. The workflow diagram is shown in Figure 4.

Z = B *f/D (1)

*H = C − Z − r* (2)

Weekly scans were conducted over the plant canopy from the same camera elevation until the plants were within the acceptable range. When the plants outgrew the minimum distance, the elevation was changed to a higher position. Each scan saved RGB images, depth maps, and disparity information with the corresponding date and time of the day. Each week, while collecting scans of the crops, the true heights of the plants were also measured as field plant heights (*FPH*s). Since all the plants were not the same height up to the tip of the plant top, multiple plant stems were measured under the device area. The maximum measurement among the recorded heights is considered the representative plant height and is used as validation.

The plant leaves in any stage of image collection were at different elevations all along the stem, so pixel-wise-calculated heights of image samples have been plotted using a violin distribution to illustrate the overall plant canopy profile and spread according to the plant age. The plant heights of segregated leaf pixels were also plotted three-dimensionally, and the height profile was mapped using a color bar for advanced interpretation. Residuals, bias, and the root mean squared error (*RMSE)* in Equation (3), and the bias-adjusted root mean squared error (*RMSE_B*) in Equation (4) were calculated to compare the accuracy with Neupane et al. [44]. Considering the manually measured field plant height (*FPH*) and image-based plant height (*IPH*), the residual is measured from the difference between the FPH and IPH, and the bias is measured as the average of the changes from *IPH* compared to *FPH*.

(3) $$\mathrm{RMSE}\;\;\sqrt{\frac{\sum_{i=1\;}^{i=n}{\left(IPH-FPH\right)}^{2}}{n}}$$

(4) $$\mathrm{RMSE}\_B\;\;\sqrt{\frac{\sum_{i=1\;}^{i=n}{\left(IPH-FPH-Bias\right)}^{2}}{n}}$$

The case study was in downtown Toronto, which had a humid continental climate and experienced changes in its temperature more rapidly between day and night, with nights exhibiting lower temperatures. The study monitored the rooftop temperature and humidity for four consecutive months—June (mild–hot to hot), July (hottest), August (hot to mild–hot), and September (mild–hot to mild–cold). The meteorological data on the ambient roof climate were collected from 1st June to 23rd September. Temperature data gathered from crop canopy levels were analyzed for diurnal variation and cooling advantages. The average cooling effects were calculated by comparing the mean daily temperatures of the control roof with the mean daily temperatures above the plant bed at 15 cm and 60 cm heights. The projected cooling advantage from the soil substrate compared to the plants has also been investigated in this study.

## 4. Results and Discussions

### 4.1. PBGR Crop Growth Monitoring

**Figure 5:**
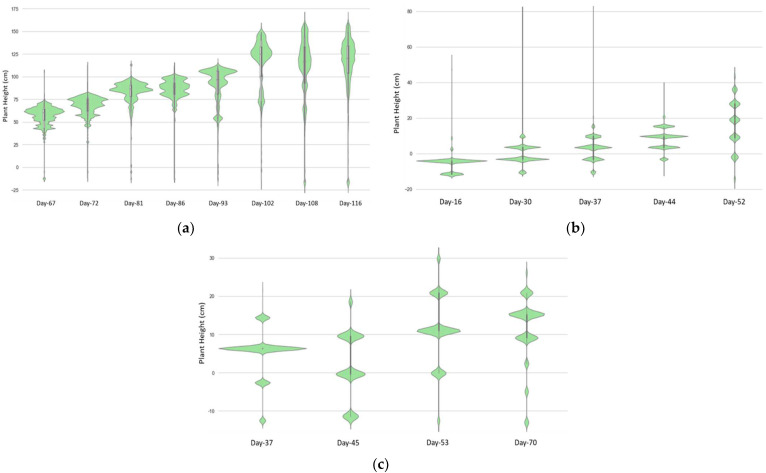
Plant height distribution in violin plots: (**a**) okra; (**b**) bush bean; and (**c**) carrot.

Plant leaves do not have the same height level; instead, they vary across the canopy based on their shape, height, angle, and orientation, and the following violin plots in Figure 5 illustrate the segmented leaf pixels that are distributed in varying height regions along the stem height. The violin distributions also show how the overall plant height increased over the weeks during monitoring, indicating the crop growth pattern. These plots simply represent the heights of the segmented leaf regions which are visible and captured from the top of the canopy and do not represent the entire plant. The occluded plant organs below the covers are not contained here. The wider section represents the distribution of the height by most of the visible leaves, and the narrower section corresponds to less visible areas. Here, the upper region of the plot represents the probable plant height which is the concerned parameter here. Among the values contained in the weekly sets of heights, the highest point of each set should be taken as the plant height. However, for this case study, the value that led to the best result with the least error was found in the 98th to 99th percentile range of each set.

**Figure 6:**
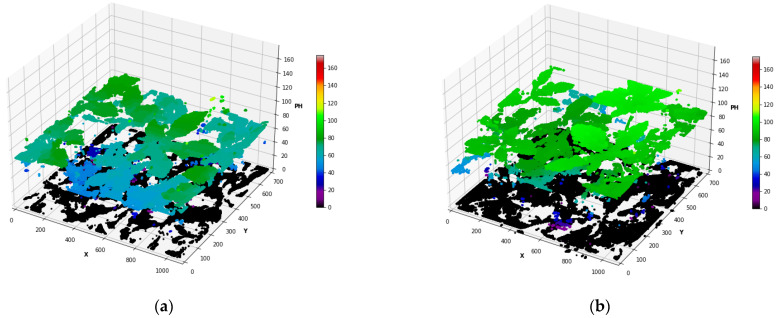
Upper canopy point cloud of okra: (**a**) 10 August 2022 (72 days of planting) and (**b**) 24 August 2022 (86 days of planting).

In Figure 5a, the weekly heights are drawn with violin plots from day 67 (5th August) to day 116 (21st September) of planting for okra. The top of the density curve and the mean height are seen increase from approximately 75 cm (day 67) to 160 cm (day 116) with a gradual upward trend of the central and top regions of the plot. It is observed from the upper region distribution of plots after August that the okra crop’s development rate was slower than before as the stem began to lose leaves and grew thinner. The fact is evident from the 2D RGB images as well. The plots also demonstrate an understanding of the leaf volume in plant stems up until day 108 and a decrease in leaf area by the end of September. The plots from day 108 and later portrays how narrow the plant profile is in the field at a mature age, having fewer captured leaves. The dynamic changes in okra plant height for the monitoring phase are noticeable here. Some points fall under zero, which are background points situated below plant bed level ‘r’, showing the point below the raised plant bed. The field of view narrows as the plants get taller, getting closer to the sensor. Plant tips or shoots can grow in all directions other than vertically, making it challenging for the lens to capture, and the sensor cannot accommodate them. This problem affected the measurement when the okra was taller than 150 cm. The wheeled cart’s highest elevation that the sensor could be positioned was 204.5 cm. The erroneous estimates of negative residuals for okra stems that were above 170 cm occurred because the tips were either only partially captured or came too close for the sensor to identify. The estimated heights measured from the okra images were used to extract the three-dimensional upper canopy construction of the monitored okra plants (Figure 6).

For the weekly height distribution of comparatively shorter bush bean plants, the plots of the exceedingly preliminary stages of the plant at day 11 present mostly outliers associated with the images. The first few days have shown high errors in extracting the height because of the field condition at image acquisition while working in very bright light. In the later days, the distribution rose higher as they matured with bushy leaves at varying heights. The maximum height values also increased showing growth over the season up to 49 cm in Figure 5b. The 98th and 99th percentiles are found as 28.78 cm and 36.87 cm in the last plot, whereas the maximum plant height of that period measured from the field that day was 33 cm. The study estimated the plant height using the bed level at 17 cm, which may have been influenced by the overall errors. Like okra, it is seen that some of the values reached the negative because as they aged, the plants plunged on the bed and went below the plant bed level around the edges of the plant bed, which impacted the plots to have negative heights. In addition to that, the plant bed level had pores and holes instead of being completely flat which impacted the plant height calculation for some points even after segmentation.

Carrot plants were quite different from the preceding two plants, in that they have small, thin structured leaves and the top growth is less as well being a root vegetable. A similar result to bush beans was found for the carrots in Figure 5c, although the plant height was higher this time. The precision could have been better if the final binary image were better segmented. The plants were increasing until day 53, reaching around 33 cm, and on day 70 (10 October), it was seen to be lower in the plots. The actual maximum plant heights on days 53 and 70 were 36 and 35 cm, respectively. The height was lower because of the wind influence. Since these were captured by late September and early October, they tumbled on each other because of occasionally high wind speeds. The violin depth distribution of both bush beans and carrots displayed discontinuities while plotted. These inconsistencies were generated from the elimination of concerned pixels during the process of segmentation and morphological noise removal, and they appeared as discrete gaps. Additionally, the result was impacted by the slumped leaves and the occlusion from varying heights of different leaves. The size of the leaves was very small, and the camera located at a high altitude resulted in multiple leaves in single pixels and thus led to an inaccurate depth calculation. In addition to the camera’s spatial resolution, the absolute inaccuracy is influenced by subtle camera movement and breeze in the field while images are acquired. The estimated height also contains some discrepancies, with a higher percentage of errors at the start of the early development of bush beans and carrots. The plants’ potential movement from one image to the next might result in an inadequate construction of depth map and disparity matrix, so the wind speed of the location is also a significant barrier in the field.

The precision of the plant height estimation method implemented in this study strongly depends on the value of camera elevation from the soil path (*C*), cart settlement, plant bed elevation over the soil path (*r*), the distance of plant canopy from the sensor lens (*Z*), the accuracy of field records, and other factors like distortion, exposure, wind, and shades. The combined effects of all these variables might alter the outcome by a few millimeters to a few centimeters. Nevertheless, observing the error percentages and residuals, the accuracy of the measurements needed to be investigated. It was observed that the dataset derived from the photos was skewed towards small- and medium-sized plants, specifically the test plants, bush beans, and carrots. These plants also exhibit higher residuals. This study considered the upper boundary range (98th, 99th, and 100th percentile) for measuring plant development since, as was previously noted, extracting the maximum height from the image produced larger outliers. Hence, it is concluded that the optimum results from each scan measured from the 98th and 99th percentiles values correspond closer to the actual measurements.

**Figure 7:**
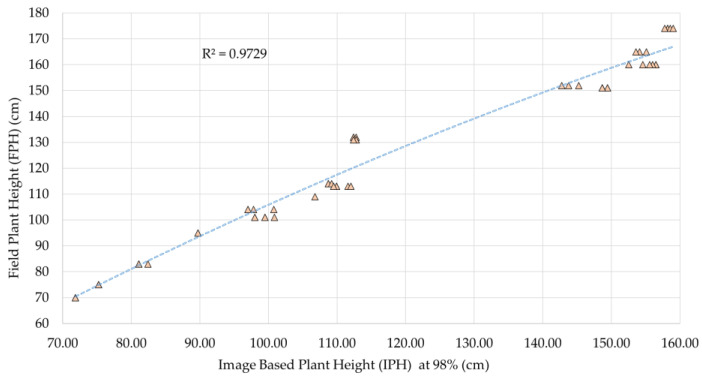
The correlation coefficient of okra FPH and IPH (98th percentile).

The *RMSE* and *RMSE_B* values measured for the 98th, 99th, and 100th percentiles of pixel heights are listed in Table 2. The results show that the *RMSE* values for carrot and bush bean ranged from 11 to 27 cm with the maximum distance from the plant bed to the camera being 2 m. Therefore, the method is found to be skewed for shorter plants. Okra plant height was measured with the lowest *RMSE* of any of the plants under observation, at 8.76 cm and 8.34 cm, respectively, based on the 98th and 99th percentiles of the height distribution. A study [44] utilized the earlier version which is Oak-D, and measured *RMSE_B* for that depth imager as 12.06 cm for indoor light and 9.69 cm in diffused sunlight. Here, in direct sunlight, both 99th and 98th percentile *RMSE_B* values are less than 7 cm, which appears to be better than the findings of Neupane et al. [44]. Petropoulou et al. deployed a RealSense camera in their study for estimating lettuce growth with an R^2^ of 0.976 [18]. In this study, the okra *FPH* and *IPH* were found to be significantly correlated, with R^2^ values for the 98th and 99th percentiles of 0.9729 (Figure 7) and 0.9711, respectively. Up to 24th August, the error percentages were less than 6% in both cases. The fact is that by week 5 of the monitoring (on 31st August), okra plants had almost reached the minimum distance of the camera that influenced some of the measured heights, showing inaccuracies as high as 19%. The problem was resolved later that week, and the error dropped to less than 9% afterward. Therefore, for taller plants like okra, the method has the potential to measure field heights provided that the relevant field condition is in favor.

### 4.2. PBGR Crop-Wise Thermal Performance

**Figure 8:**
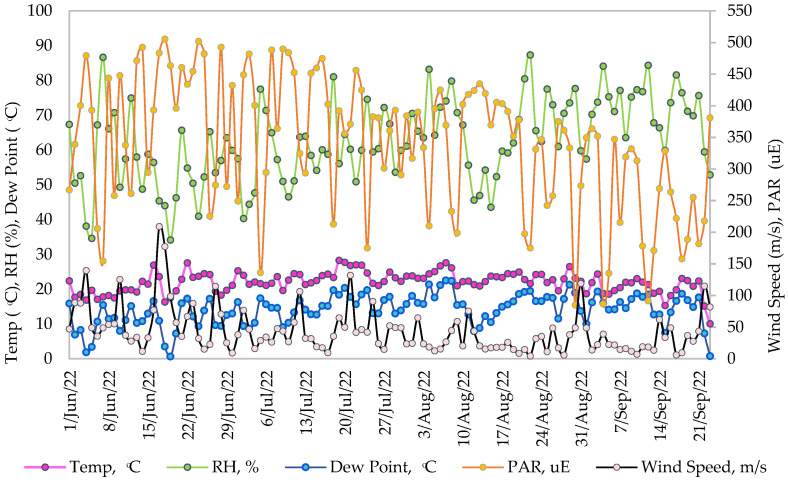
Mean daily meteorological parameters of the rooftop farm (June to September).

The average climate around Toronto around June, when the monitoring was started, was moderately hot, followed by the hottest month, July, and then the moderatelywarm weather that was observed in August, with gradually decreasing temperatures in September. The warmest period was monitored from late June to early August with maximum temperatures frequently topping 30 °C. Figure 8 portrays the graphical representation of the mean daily (24 h average) temperature, relative humidity, dew point, wind speed, and photosynthetically active radiation (PAR) for the monitoring period. Table 3 lists the statistics of the climate variables monitored on the roof in this growing season. The maximum temperature recorded was 35.80 °C in June, 34.44 °C in July, and later dropped gradually to 32.12 °C in August and 28.69 °C in September. The monthly mean daily temperature was 20.55 °C in June, 23.53 °C in July, 23.43 °C in August, and 20.09 °C in September. The relative humidity of the roof was lower at the start of monitoring with a mean of 55.70% in June and increased up to 71.68% in September, with occasional maximums surpassing 60% over the summer. The mean dew point increased from June to September depicting the amount of moisture in the air. Maximum PAR was recorded in July as the intensity and active hours of the sun were long in this month.

**Figure 9:**
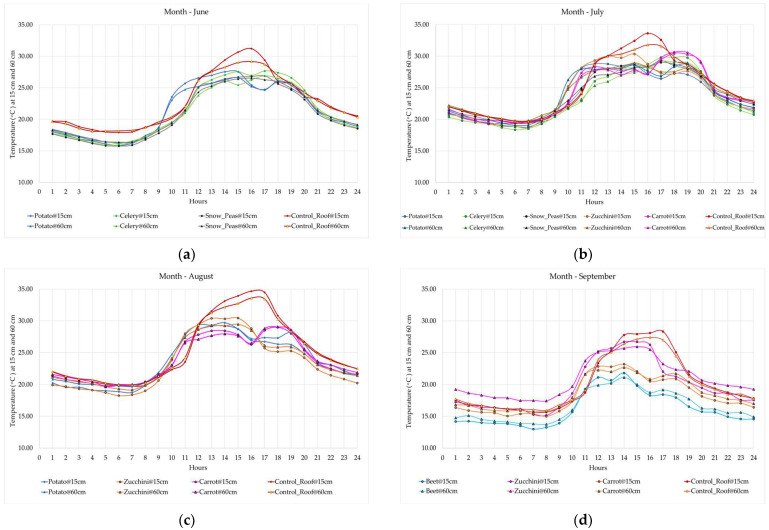
Plant-wise monthly diurnal temperature variation at 15 and 60 cm above the plant bed: (**a**) June; (**b**) July; (**c**) August; and (**d**) September.

The daily atmospheric temperatures demonstrate high variations between day and night in the study region. The daily high and daily low temperatures vary each month, so the study explored the month-wise diurnal variation among the crops. This is a good indicator of microclimate over a shorter span of period and provides better insights into the observed plants’ performance at different hours of the day. In the diurnal (mean hourly) temperature profiles shown in Figure 9, the concrete (CR) showed the most discernible temperature diurnal change over 24 hours. During summer, the concrete absorbs heat as the day starts and accumulates it, raising the temperature until evening. Later, it releases the heat back and cools down by the night’s end. The vegetated roofs (PBGR) gained heat from the start of the day until noon.

The PBGR showed lower temperatures than the concrete from noon onwards. This contrasts with the green roof study [50] where the maximum green roof cooling occurred during nighttime. As the day started, both the CR and PBGR started to absorb heat from 8 a.m. after experiencing cold at night. From 8.30 a.m. to 12 p.m., some plants did not show any cooling effect; instead, they sustained elevated temperatures compared to the CR at both 15 and 60 cm, similar to previous studies [57,66]. The PBGR absorbed this heat in summer through the soil substrate and raised the temperature and humidity of the surrounding microclimate. However, the rate of temperature gain is higher near CR and lower near the PBGR partially because of its green-spectrum reflectance capacity as well as watered media and primarily because of its thermal capacity. As the air over the PBGR gets heated, plants attempt to cool the area through evaporation and transpiration to adjust to this increased heat. Thus, during hot summer days, the PBGR could reduce the amount of heat conducted from the concrete roof and provide insulation against the scorching sun. At night, the CR surface cooled from releasing heat, whereas the PBGR did not lose much compared to that. The phenomena can be attributed to the fact that plants used their latent heat to adjust the temperature drop by reducing respiration and photosynthesis as it got cold on nights. Therefore, the PBGR did not emit as much heat as the CR and hence can contribute to saving energy. It showed a slower heat gain, resulting in expected cooling effects during the afternoon.

The greatest diurnal temperature attenuation was observed by beet (9.91 °C) in the afternoon, but this was monitored in late September and could be impaired by colder days. From full month monitoring, zucchini (8.78 °C, at 4 p.m.) and carrot (8.37 °C, at 3 p.m.) achieved high day cooling in August. Potato and carrots attained maximum summer cooling during June and July. Snow peas experienced consistently lower temperatures at 15 cm than concrete throughout the season. Crops showed maximum cooling happening after 3 p.m. June–July and 4 p.m. in August–September at both 15 and 60 cm. Morning (primary) and evening (secondary), two warming peaks were observed in some crops in June, July, and August, with potatoes acquiring temperature earlier in June and celery showing evening warming effects. In September, zucchini and carrot showed warmer mornings, with secondary warming effects only observed in zucchini. Both the CR and PBGR cooled down at night, with 15 cm PBGR cooling up to 3.83 °C by beet (9 pm in September), 2.61 °C by zucchini (8 p.m. in August), and 2.48 °C by snow peas (1 a.m. in June), with less variation suggesting a greater insulating capability. At 60 cm height, daytime maximum cooling was recorded from zucchini (7.33 °C) at 4 p.m. in August and 2.2 °C by celery at 5 a.m. in June. The impact of wind is minimal on the ground, but it becomes more prominent above the canopy layer [67]. The canopy surface weakens wind turbulence, preventing hot air above the canopy from mixing with the air below, and keeping it cooler [53].

**Figure 10:**
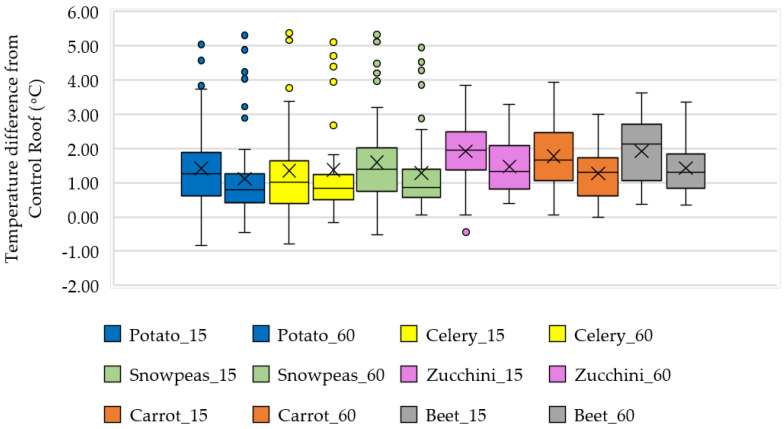
The plant-wise cooling effect at 15 and 60 cm above plant beds.

Since each crop is different with varying canopy coverage, leaf size, and density, the cooling effect varies depending on each species. Dense and bigger leaf regions are expected to lower the temperature by providing shade and high evapotranspiration. The mean daily cooling impact of the monitored crops is shown in Figure 10 with the box-whisker plot. Positive temperature differences show greater cooling by causing the PBGR temperature to fall relative to the CR. Maximum daily cooling at 15 cm was observed from celery (up to 5.37 °C), snow peas (up to 5.33 °C), and potato (up to 5.03 °C). At 60 cm, the maximum cooling was recorded from potato (up to 5.30 °C) and celery (up to 5.11 °C). Celery had a consistently dense canopy until it was harvested. Potatoes, with their thick, bushy, and leafy canopy, had an extended cooling range, but during their mature phase, reduced leaf and shading was observed. Snow peas were accompanied by cilantro, with dill plants in between, having a blended effect on the overall cooling. However, the highest mean cooling of 1.92 °C was achieved by beet (median 2.13 °C) and zucchini (median 1.94 °C) at 15 cm. Beet presented a considerably high mean cooling due to its broad leaves, a moderately dense canopy resulting in its ability to reflect sunlight and draw additional water from the soil. Celery and potato showed the greatest diurnal heat fluctuation among the crops.

The substrate temperature can present how effectively a building’s roof is insulated from the external temperature. The soil substrate acts as temperature retainer media in parallel to vegetation, and in a PBGR, the plant’s productivity can be negatively impacted by excessive heat stress, causing flower buds to fall off the plant. In the presence of adequate or excessive moisture content during warm temperatures, soil releases water vapor through evaporation and hence contributes to humidity improvement, which facilitates plant growth. Temperatures above damp soil could differ up to 5 °C at 1 m above the ground [53], testifying to the significance of cooling effect.

**Figure 11:**
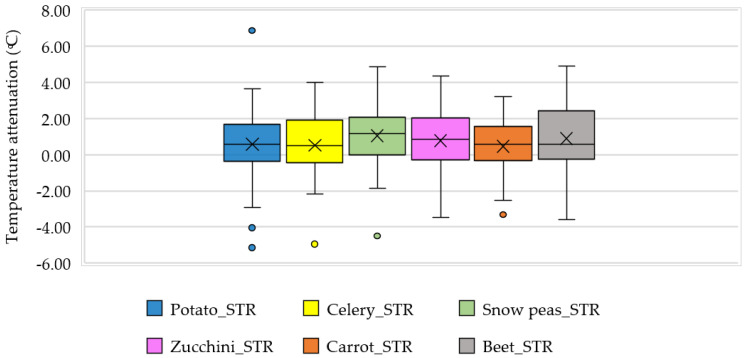
Plant-wise substrate temperature attenuation compared to the plant canopy at 15 cm.

The study explored the PBGR substrate temperature variation in comparison to the temperature that plants experienced just above the soil. Figure 11 depicts the box-whisker plot of the mean temperature drops in substrates compared to the air temperature at 15 cm above soil, with positive temperature meaning further cooling from the plant layer. The plot showed that the mean temperature decline in the snow pea substrate is maximum with a mean attenuation around 1.05 °C (median 1.19 °C), followed by beet at 0.90 °C (median 0.57 °C). Zucchini showed a comparable mean cooling of 0.77 °C (median 0.84 °C), potato and celery ranged at 0.59 °C (median 0.57 °C) and 0.52 °C (median 0.49 °C), and carrot showed the least reduction of only 0.47 °C (median 0.58 °C). There were some events where the substrate was higher than the corresponding temperature at 15 cm that was observed from the box plots, which could happen due to the antecedent dry condition of soil with air pockets saturated with warm air. This elevated temperature fluctuation was higher for potatoes (5.13 °C), celery (4.93 °C), and snow peas (4.50 °C). These higher temperature variations happened in the substrate due to insufficient moisture content [51], as a moist, intensive roof system would retain heat throughout the day on sunny days and prevent temperature drops at night [67]. The air beneath the canopy also remains cool and humid, supplemented by evapotranspiration [53] and irrigation.

## 5. Conclusions

This case study demonstrated the potential for the new depth camera to be used in field settings for crop growth monitoring. As more depth cameras arrive on the market, it is crucial to investigate their capabilities and performances to determine the optimal approach and fit. The majority of these devices are made for indoor usage, but there is a possibility that they may be upgraded for everyday use with a permissible range of errors that would reduce the amount of labor-intensive monitoring required. The quantification of the plant growth and development in a working urban farm (instead of the small test beds in the literature) will significantly help comprehend the potential plant response in the urban environment and guide future planting actions. However, the study accuracy was influenced by subtle camera movement and breeze in the field while images were acquired. The estimated height also contained some residuals, with a higher percentage of errors at the start of the early development of bush beans and carrots. Crop movement from one image to the next might result in an inadequate construction of a depth map and disparity matrix, so the wind speed of the location is also a significant barrier in the field. The detectable pixels and their disparity from the top determine how far the canopy extends from the lens. Due to the nature of the experimental settings, the points that were determined to be closest (the tallest plants) from an image and the point where the *FPH* was measured were difficult to match.

Images could not be obtained under constant light circumstances due to the limited schedule of the fully operational farm and the rapid shift in daylight later in the season. Sunny daylight and shade substantially impacted the estimation method, lowering the precision based on which phenotypic features were computed. The segmentation was also significantly impacted in the presence of shadowed regions from the platforms and nearby plant stems and leaves, and these areas were deleted when the foreground was partitioned from the background. Therefore, the highest point of the canopy would not be present in the foreground if it had been shaded. The results for small plants could be enhanced by adjusting the camera height to a shallower depth with a fill flash for consistent light conditions. Since segmentation was carried out using built-in Python tools in an automated manner, the actual closest points may be affected or eliminated. To further enhance the strategy, an improved segmentation algorithm should be explored. The system had to be given 3–4 min of buffer time between the scans to let the system match the runtime lag. Otherwise, the preceding scans have a chance to get overwritten by the recent ones. The system should be tested by connecting to a mobile host, and further information about the target plant’s environments such as the correlations between crop development characteristics and environmental factors like direct sunlight, ambient temperature, and humidity could be incorporated.

Another significant attribute of this research is understanding how the environmental parameters (plant level temperature and substrate temperature) vary in such roofs and correspond to the cumulative benefits, rooftop performance, and plant growth. The thermal advantage of the productive roof was demonstrated by the fact that such configurations had lower daytime and nighttime temperatures than concrete. The attenuation of temperature is more evident on the days with high temperatures. The overall cooling effect of the case study was maximum during the afternoon from 12 to 3 p.m. during the summer. In the late afternoon, some crops exhibited the warming effect which lasted until early evening. The concrete and plant layers both cool down in the evening after sunset, releasing heat into the environment. An exception is observed for zucchini plants in September with a long warming effect at night. The substrate temperature was lower than the plant layer temperature at 15 cm. The growth media’s evaporation and heat mass capacity kept it lower in the presence of moisture content. None of the crops were subjected to severe nutritional or water stress during the study. More crop species should be tested for their thermal performance to aid crop planners. The measurement of evapotranspiration can improve the understanding of plant thermal response. Future research is advised to investigate how various crop species might provide the projected cooling benefit in extreme climatic conditions.

Although low-cost IoT sensors are now built to last for several years, their effectiveness in continuous monitoring should be evaluated by future studies. The image sensor in this study required a power connection supplied by the host battery while the scans were being performed. The LoRaWAN temperature sensors were battery-powered and worked for two growing seasons without battery replacement. However, the LoRaWAN soil moisture sensor needed frequent battery replacement, and it was identified that the resolution of the monitoring interval had a significant impact on this, as frequent data transmission uses more power. A high communication range can lead to a slow data flow rate. The transmission range for maintaining wireless connectivity is also important for this. Further research is required to assess the power consumption and supply solutions of such IoT networks. Also, the data collected by the TTN sometimes were incapable of communicating to the ThingSpeak’s channels. This resulted in missing data if it were not periodically inspected during the monitoring.

The automated monitoring system designed in this study can be a functional tool for productive blue-green roof operators and researchers to monitor and quantify in-field crop growth. It can be beneficial to operate rooftop farms in real time without much effort. There are prospects to improve the developed system using low-cost image sensors and help recognize the difficulties that are likely to occur in terms of field deployment, as well as the technical limitations of such sensors in continuous monitoring and operation.

## Figures and Tables

**Table 1:** Monitored crop species and families for plant growth and thermal performance.

	Crops	Family [58]
Plant Growth Monitoring	Okra	Malvaceae (mallow family)
	Bush Beans	Fabaceae (pea family)
	Carrot	Apiaceae (parsley family)
Cooling Effect monitoring	Potato	Solanaceae (nightshade family)
	Celery	Asteraceae (daisy family)
	Snow peas	Fabaceae (pea family)
	Summer Squash	Cucurbitaceae (gourd family)
	Carrot	Apiaceae (parsley family)
	Beet	Amaranthaceae (flowering plant)

**Table 2:** RMSE and RMSE_B of measured plant heights.

	98 Percentile		99 Percentile		100 Percentile	
	RMSE	RMSE_B	RMSE	RMSE_B	RMSE	RMSE_B
Okra	8.76	6.19	8.34	6.23	15.34	14.99
Bush Bean	11.69	5.33	11.53	9.17	27.06	24.06
Carrot	12.95	5.16	11.43	20.12	26.30	37.07

**Table 3:** Mean, maximum, and minimum of climatic parameters (June to September).

		June	July	August	September
Temperature °C	Mean	20.55	23.53	23.43	20.09
	Max	35.80	34.44	32.12	28.69
	Min	10.30	15.32	15.20	7.24
Relative Humidity %	Mean	55.70	59.69	66.08	71.68
	Max	95.50	96.20	92.40	95.80
	Min	15.10	27.80	24.00	36.70
Dew Point °C	Mean	10.77	14.84	16.35	14.66
	Max	22.89	22.92	25.05	21.75
	Min	−6.20	5.78	0.42	−0.23
Wind Speed m/s	Mean	0.66	0.44	0.30	0.31
	Max	6.00	4.00	3.00	3.50
	Min	0.00	0.00	0.00	0.00
Photosynthetically Active Radiation μmoles s^−1^ m^−2^	Mean	383.96	373.55	330.67	246.50
	Max	2226.00	2301.00	2259.00	1974.00
	Min	1.00	1.00	1.00	1.00

## Data Availability

Available from the corresponding author upon reasonable request.
